# Visual contextual perception and user emotional feedback in visual communication design

**DOI:** 10.1186/s40359-025-02615-1

**Published:** 2025-03-28

**Authors:** Jiayi Zhu

**Affiliations:** Academy of Arts, Qujing Normal University, Qujing, Yunnan 655011 China

**Keywords:** Multilayer CNN, Dual attention mechanism, Holistic and local feature, Sentiment analysis, Visual communication

## Abstract

**Background:**

With the advent of the information era, the significance of visual communication design has escalated within the realm of increasingly prevalent network applications. Addressing the deficiency observed in prevailing sentiment analysis approaches in visual communication design, which predominantly leverage the holistic image information while overlooking the nuances inherent in the localized regions that accentuate emotion, coupled with the inadequacy in semantically mining diverse channel features.

**Methods:**

This paper introduces a dual-attention multilayer feature fusion-based methodology denoted as DA-MLCNN. Initially, a multilayer convolutional neural network (CNN) feature extraction architecture is devised to effectuate the amalgamation of both overall and localized features, thereby extracting both high-level and low-level features inherent in the image. Furthermore, the integration of a spatial attention mechanism fortifies the low-level features, while a channel attention mechanism bolsters the high-level features. Ultimately, the features augmented by the attention mechanisms are harmonized to yield semantically enriched discerning visual features for training sentiment classifiers.

**Results:**

This culminates in attaining classification accuracies of 79.8% and 55.8% on the Twitter 2017 and Emotion ROI datasets, respectively. Furthermore, the method attains classification accuracies of 89%, 94%, and 91% for the three categories of sadness, surprise, and joy on the Emotion ROI dataset.

**Conclusions:**

The efficacy demonstrated on dichotomous and multicategorical emotion image datasets underscores the capacity of the proposed approach to acquire more discriminative visual features, thereby enhancing the landscape of visual sentiment analysis. The elevated performance of the visual sentiment analysis method serves to catalyze innovative advancements in visual communication design, offering designers expanded prospects and possibilities.

## Introduction

Individuals engaged in the cognitive process, with a primary focus on self-perception, utilize visual communication of information to discern and comprehend the world. The integration of visual context perception [1] in graphic creative design facilitates the achievement of distinctive and personalized developmental objectives in graphic design. Emphasizing a comprehensive understanding of the fundamentals of graphic design, the incorporation of the visual context perception principle enhances original design concepts, imbuing graphic design with a renewed significance. Visual context perception has demonstrated newfound vitality in graphic creative design, prompting designers to contemplate its optimal application. Proficiency in visual context perception holds both theoretical and practical significance for the advancement of its principles. The synergy between visual context perception principles and the common development of graphic creative design serves as a substantial enrichment, ensuring the principles remain dynamic and vigorous in future developments.

The current era benefits from groundbreaking advancements in computer technology and deep neural network technology, propelling artificial intelligence research to unprecedented heights [2]. In this evolving landscape, an increasing number of researchers direct their attention to the user's emotional dynamics under visual context perception as a derivative of intelligence. The exploration of affective computing seeks to restore bio-emotional intelligence. Visual sentiment analysis [3], an integral component of affective computing, involves predicting psychological changes in individuals upon viewing an image through the user's visual situational awareness and emotional feedback measurements. This predictive capability allows for a certain degree of control over the user's emotional responses under visual situational awareness, holding significant application prospects across diverse fields.

Emotion classification aims to identify and analyze emotional tendencies within media such as text and images, while visual communication design strives to convey specific information and emotions through visual elements. Both disciplines share the goal of achieving precise emotional transmission and perception. In the creative process of visual communication design, consideration must be given to how to evoke emotional resonance in viewers, which is essentially an implicit process of emotion classification. Designers must determine which visual elements will elicit specific emotional responses to inform their designs. Similarly, the results of emotion classification tasks can guide visual communication design, helping designers better understand the emotional needs of their target audience. With technological advancements, emotion classification techniques are increasingly being applied in the field of visual communication design. For instance, by analyzing users' emotional feedback on design works, designers can optimize their designs and enhance their appeal. Additionally, principles and methods from visual communication design can inspire and serve as a reference for emotion classification tasks.

As the demand for emotional analysis in visual communication design grows, in recent years, visual sentiment analysis has garnered considerable attention from researchers globally; however, several unresolved challenges persist. The primary issue stems from the inherent abstractness of visual emotion recognition in images, in contrast to the more tangible target recognition task. The crux of the matter lies in the extraction of discerning visual features [4]. Although prior studies have integrated deep learning techniques [5] with visual sentiment analysis, yielding noteworthy results, certain limitations endure. Existing methods typically extract features from the entire image, neglecting the crucial local regions and overlooking multi-level features, thus failing to fully exploit the spatial and semantic dimensions of the information. This deficiency leads to a suboptimal expressive power of the features. To address this, the present study leverages the complementary nature of multilevel features to augment their efficacy. It introduces the DA-MLCNN method, grounded in dual-attention multilevel feature fusion, to acquire more distinctive visual features and enhance the sentiment classification performance. The specific contributions of this paper are outlined below:

1. Constructing Feature Enhancement and Local Region Optimization Strategy. This innovative approach focuses on the critical local region information within images, leveraging the complementary strengths of multi-level features. Through a meticulously designed local region selection mechanism, it precisely captures image details rich in emotional information. Simultaneously, the fusion and enhancement techniques for multi-level features significantly boost the discriminability and robustness of feature representations. This strategy is novel in the field of visual emotion analysis, providing a more precise and abundant feature foundation for emotion recognition.
2. Developing a Multi-Dimensional Attention-Guided Deep Feature Extraction Framework. This architecture automatically extracts multi-channel, multi-level deep features from images. By incorporating advanced attention mechanisms, it applies spatial attention weighting to low-level features to highlight emotionally relevant spatial regions, while utilizing channel attention weighting for high-level features to emphasize the contributions of critical channels. This dual attention-guided feature extraction approach significantly enhances the specificity and effectiveness of feature representations, offering robust support for emotion classification tasks.
3. Crafting an Efficient Fusion Strategy to Construct Discriminative Emotion Features. This strategy integrates low-level detailed features, enhanced by attention mechanisms, with high-level abstract features at the feature fusion layer, achieving information complementarity and enhancement. The resulting discriminative features are tailored for training emotion classifiers.

This paper delineates the current state-of-the-art in visual sentiment analysis, encompassing both local regions and deep learning methodologies in Section 2. Section 3 introduces DA-MLCNN, a novel visual sentiment analysis method grounded in dual-attention multilayer feature fusion. Section 4 is dedicated to elucidating experimental results, scrutinizing scheme performance, and conducting comparative analyses with classical approaches. Ablation experiments are further employed to dissect the individual contributions of each model module. The final section investigates the enhanced performance of the DA-MLCNN model on visual communication. Section 5 concludes with a comprehensive summary, delving into the performance of the DA-MLCNN model constructed in this study and its implications for visual communication.

## Related works

### Visual communication based on emotional recognition

Visual symbols serve as a powerful means for designers to communicate design ideas with enhanced effectiveness and conciseness, often surpassing the communicative impact of verbal text. Exceptional design endeavors exhibit a distinctive essence, encapsulating unique thoughts and emotions within the creative output. In visual design activities, the process of image creativity involves not only the redesign and re-creation of images by the designer but also the expression of personal emotions [6]. Designers leverage their works to convey emotional elements to the audience, fostering an intuitive connection where viewers discern their emotional fluctuations, thus reinforcing the emotional bond between the designer and the audience.

Traditional emotion recognition in visual communication typically relies on manually designed base features for classification. Commonly employed features include Gabor wavelet, scale-invariant feature SIFT, gradient direction histogram HOG, and local binary histogram LBP. Subsequently, statistical models such as HMMS, GMMS, SVM, and others are utilized for classification [7]. A framework proposed in literature [8] utilizes mid-level features to predict the emotional state of a picture and adjust visual product design architecture based on user emotional changes. Another approach, presented in literature [9], introduces a face expression recognition method grounded in dynamic coding of expressions, utilizing GMMs to model spatio-temporal features of screenshot videos depicting emotional changes. Additionally, literature [10] introduces a multi-support vector neural network incorporating the whale-grasshopper optimization algorithm, combined with a classifier for visual emotion classification. Recognizing emotional changes provides valuable insights into the audience's reaction to design works, enabling designers to convey corresponding emotional responses through visual elements, captivating the audience's attention and eliciting empathy.

The artificially designed features are often based on specific datasets or assumptions, which can significantly compromise the recognition performance of the model when confronted with varying environments, lighting conditions, cultural backgrounds, or individual differences. For instance, a feature extractor designed for clear images in bright environments may struggle to effectively extract crucial information from low-light, blurry, or occluded video frames, leading to recognition errors. Human emotional expressions are incredibly complex and multifaceted, manifested not only through facial expressions but also encompassing voice, posture, context, and other dimensions. Traditional approaches tend to focus solely on the facial expression dimension, overlooking other vital cues, making it difficult to comprehensively capture and understand intricate emotional states. Furthermore, even facial expressions themselves encompass intricate nuances such as micro-expressions and eye contact, which necessitate more advanced feature extraction and fusion techniques for accurate capture.

### Visual sentiment analysis based on deep learning

The burgeoning field of artificial intelligence has propelled deep learning into the forefront of research endeavors. Deep learning models, employing a strongly supervised learning approach, possess the capability to autonomously discern discriminative features from copious amounts of labeled raw data. This obviates the need for intricate feature engineering, yielding more resilient deep features compared to traditional manual extraction methods. Notably, the CNN stands out for its significant advantages in visual tasks. Numerous researchers have harnessed deep learning models to tackle visual sentiment analysis tasks, demonstrating their superiority over conventional approaches through an extensive array of experiments. As elucidated in literature [11], CNNs exhibit the capacity to apprehend information regarding objects within a scene during a scene recognition task. The study further establishes that the same network can seamlessly execute both scene recognition and object localization in a singular forward pass, underscoring the learning prowess of CNNs in localized regions. Additionally, literature [12] posits that emotions are triggered by specific regions, introducing the Emotion ROI dataset. Each image in this dataset encompasses multiple annotator-drawn rectangular boxes signifying regions of attention for the viewer. The underlying assumption is that the influence of each pixel on evoking emotion is proportionate to the number of drawn rectangles covering that pixel.

Literature [13] amalgamates the principles of convolutional neural networks and visual sentiment to advance visual sentiment analysis, presenting an evolved iteration akin to SentiBank [14]. Building on the foundation of CNN architecture, literature [15] introduces the Progressive Convolutional Neural Network (PCNN) to showcase the heightened performance capabilities of convolutional neural networks. Subsequently, literature [16] refines the network architecture using a step-by-step training strategy tailored for large and noisy training datasets, yielding further improved results. Following this trajectory, a multitude of deep neural networks, renowned for their superior performance in processing expansive datasets, have emerged as focal points of research in the field of vision. In literature [17], decoder and fully-connected layers are integrated with the fine-tuned VGG-16 network [18], trained on the Emotion ROI dataset. This integration achieves both emotion region detection and emotion classification. Similarly, literature [19] employs Alex-Net and Res-Net for visual sentiment analysis, enhancing the parameters of the Focal loss function to address the sample imbalance issue within the training set data. Deep Convolutional Neural Networks exhibit robust feature extraction capabilities and demonstrate a pronounced advantage in abstract feature extraction, particularly for features challenging to articulate definitively. Moreover, the potential for improvement is considerable, given the significant impact of alterations in network structure on performance variation.

Acknowledging the historical success of traditional image feature methods, researchers are now exploring the integration of these methods into CNNs. In literature [20], a fusion of traditional sentiment analysis techniques and CNNs incorporates parameters such as gradient histogram, color histogram, energy, and variance of wavelet decomposition, three-channel histogram similarity, as well as average luminance, saturation, and hue of an image as low-level features. However, direct fusion results in feature redundancy. Because there may be information overlap between these features, it not only increases the complexity of the model, but also reduces the computational efficiency, making the model require more time and resources in the training and reasoning process. Traditional image features are often designed manually for specific tasks or data sets, and they differ in nature from the hierarchical features learned automatically by CNN. Direct combination of the two may face the problem of feature compatibility, that is, the scale, distribution or semantic differences between different features may lead to poor fusion effect and affect the final performance of the model. Furthermore, literature [21] introduces the Object Semantic Attention Network, leveraging a convolutional neural network for global feature extraction and subject targeting on the local branch. However, the extraction of subject objects on the local branch may be limited by the ability of traditional image processing methods, which can not accurately capture the complex relationship between objects or context information. At the same time, the fusion between global and local features may not be efficient enough, resulting in information loss or redundancy.

To address visual sentiment analysis challenges, the prevailing research direction involves specialized methods utilizing deep convolutional neural networks as the core, complemented by traditional sentiment theory. This approach harnesses the automatic learning capabilities of deep convolutional neural networks for abstract features, while concurrently considering human comprehension of the cognitive process underlying emotional experiences. As a result, this methodology exhibits distinct advantages in addressing visual emotion analysis problems.

## Model design

**Fig. 1:**
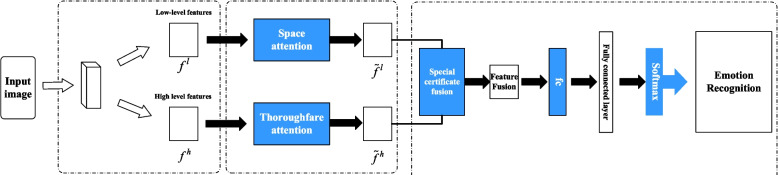
Model frame drawing

The schematic representation of the DA-MLCNN model, a visual sentiment analysis model predicated on dual-attention multilayer feature fusion, is depicted in Fig. 1. The model comprises three principal components: multilayer CNN feature extraction, dual-attention mechanism, and sentiment classification through attention feature fusion.

CNN is widely used in image processing because of its powerful feature extraction capability. The multi-layer CNN structure can abstract image information layer by layer, from low-level edges and textures to high-level shapes and semantics. This hierarchical feature extraction method helps to capture the information of images at different scales, and provides a rich feature basis for subsequent sentiment analysis. To augment the discriminative quality of image features, the image undergoes initial input into a multilevel CNN model characterized by a complex structure, facilitating the extraction of features at various levels. These encompass low-level features derived from the shallow layers of the CNN and high-level features extracted from deeper layers.

The attention mechanism simulates the ability of the human visual system to automatically focus on key areas when processing complex scenes. In visual emotion analysis, the emotion expression of different images may focus on different spatial areas or channel features such as color and texture. Therefore, the introduction of dual attention mechanism can more accurately locate the key part of the image expressing emotion, and improve the discriminative feature representation.

By integrating the output of spatial attention and channel attention module, the advantages of both can be synthesized to form a more comprehensive and discriminative feature representation. This fusion method helps the model to capture the emotion information in the image more accurately in the emotion classification. Therefore, the extracted low-level features are channeled into a spatial attention module, strategically designed to concentrate on image portions that more accurately reflect emotion, thereby accentuating these pivotal regions. Concurrently, the high-level features are input into the channel attention module, which serves the purpose of sieving out the most pertinent channel features to optimize feature selection. Ultimately, the outputs from the spatial attention module and the channel attention module are fused to craft a discriminative feature representation, thereby enhancing the accuracy and efficacy of the classification process.

### Multi-Layer CNN feature extraction

**Fig. 2:**
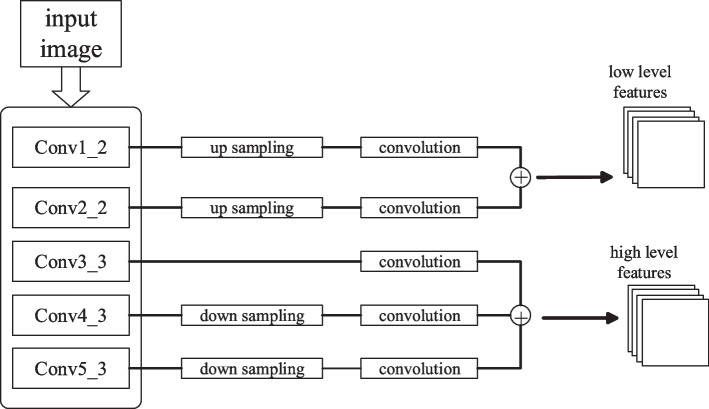
Multi-layer CNN feature extraction

This paper employs VGGNet-16 as the foundation for multilayer feature extraction. VGGNet-16 comprises five convolutional blocks and three fully connected layers, with the convolutional blocks incorporating convolutional and pooling layers, as illustrated in Fig. 2 Building upon the VGGNet-16 architecture, the features output from each layer are standardized to the same size using various sampling methods.

To transform VGGNet-16 into a FCN, I eliminated the last two fully connected layers from the original architecture and substituted them with convolutional layers featuring 1 × 1 kernels. This modification not only diminishes the parameter count but also aids in preserving the spatial integrity of information, enabling the network to output feature maps with the same spatial dimensions as the input image. This facilitation streamlines subsequent feature fusion and upsampling processes.

To acquire feature maps with higher resolutions, I specifically altered the stride settings of POOLING 4 and POOLING 5 in VGGNet. Originally, both of these pooling layers had strides of 2, significantly reducing the spatial dimensions of the feature maps. By decreasing their strides from 2 to 1, I retain more intricate details without significantly augmenting computational overhead, which is paramount for precise identification of salient regions. To harness the full potential of image features across various scales, I appended multiple convolutional layer branches following each of the first four pooling layers (POOLING 1, POOLING 2, POOLING 3, POOLING 4) in VGGNet. Each branch comprises convolutional layers with diverse configurations, varying in kernel sizes, numbers, or strides, to produce feature maps with distinct receptive fields and resolutions. These multi-scale feature maps encapsulate both local detailed information and broader contextual cues, providing a rich feature representation for subsequent tasks such as saliency detection or classification.

The saliency map is normalized by a Sigmoid activation function to produce a probability distribution of the saliency of the image $A_{s} \in {\mathbb{R}}^{W \times H}$, calculated as:

1 $$A_{s} = Sigmoid(S)$$

Additionally, the feature representation of the saliency region of the image is ultimately acquired by multiplying the probability distribution of the image's saliency with the overall features of the image. This involves weighting the feature representation of the saliency region to emphasize its significance in the overall feature representation of the image $F_{s} \in {\mathbb{R}}^{W \times H}$.

Let $\oplus$ denote the multiplication between the corresponding elements is calculated as:

2 $$F_{s} = A_{s} \oplus F$$

Furthermore, for each channel's feature maps, the average value is computed, and this average value is employed to substitute each channel's feature map. Consequently, this transformation results in the conversion of the feature $F_{s}$ into a feature vector denoted as v, i.e., *V* = *GAP*($F_{s}$). For each element of the feature vector v $v_{i} ,i \in \{ 1,2,...,C\}$, it is computed as:

3 $$v_{i} = \tfrac{1}{W \times H}\sum\nolimits_{m,n} {f_{i} (m,n)}$$

where $f_{i} (m,n)$ denotes the value of the (m,n) position element of the i-th feature map of the feature $F_{s}$. The feature vectors are then input into a fully connected layer for classification. Each final output classification result corresponds to a weight parameter in the fully-connected layer. Let c denote the final output category of the fully-connected layer. The class activation mapping for this category is calculated as follows:

4 $$M_{c} = \sum\limits_{i = 1}^{c} {w_{i}^{c} f_{i} }$$

where $w_{i}^{c}$ denotes the corresponding weight of the fully connected layer, and $f_{i}$ denotes the i-th feature map of $F_{s}$. The loss function for this process, i.e., CAM loss, is computed as follows.

5 $$L_{CAM} = - \tfrac{1}{N}\sum\limits_{i = 1}^{N} {\sum\limits_{c = 1}^{k} {\Gamma (y_{i} = c)\log v_{c} } }$$

where $\Gamma (s)$ denotes the condition function and $y_{i}$ denotes the real label of the image. Updating the weights of the convolution kernel facilitates the extraction of activation regions specific to different emotion categories within the feature map.

### Dual attention mechanisms

**Fig. 3:**
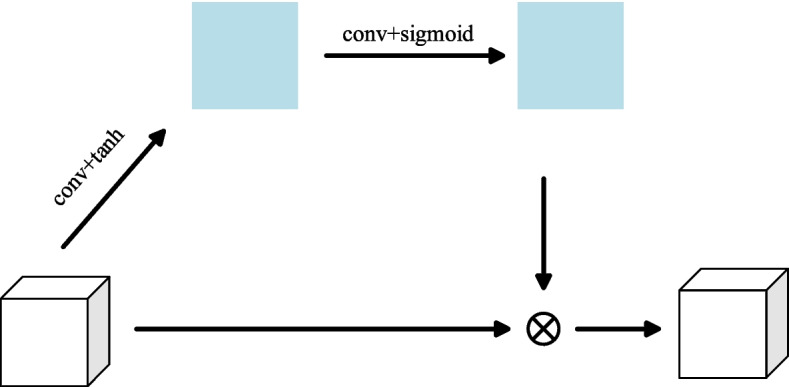
Spatial attention mechanism

Relying solely on global image feature vectors for image emotion classification may not yield optimal results. Recognizing the significance of local regions in conveying the overall emotional expression of an image, specific regions such as those containing flowers and smiley faces are incorporated into the spatial attention mechanism. This mechanism is trained to identify local regions in the image that emphasize emotion, assigning higher weights to enhance the representation of features in the spatial domain of the image. The structural details of the spatial attention module are illustrated in Fig. 3.

The low-level features $f^{l}$ are dimensionalized by a convolutional layer with a convolutional kernel size of 1 × 1, and then the feature mapping M with a size of $W \times H$ is generated by the tanh activation function, and then M is passed through the convolutional layer and the sigmoid activation function to generate the spatial attention weights $sa$, which are calculated as:

6 $$M = \tan ({\text{co}} nv_{1} f^{l} )$$

7 $$sa = sig{\text{mo}} id(conv_{2} M)$$

where $conv_{1}$, $conv_{2}$ denote the corresponding convolution operations. The final weighted feature output is:

8 $$\tilde{f}^{l} = sa \otimes f^{l}$$

where $\otimes$ denotes elemental multiplication.

The extracted features in CNN comprise different channel features with varying semantic information. Consequently, the importance of different feature channels in sentiment classification varies. Current visual sentiment analysis methods based on deep learning tend to treat each channel feature equally, imposing limitations on sentiment classification performance. To address this, the channel attention mechanism is employed to prioritize important channel features while filtering out irrelevant ones, essentially recalibrating the features. In this study, the author introduce the channel attention mechanism to assign greater weights to channel features that exhibit high responsiveness to sentiment, thereby enhancing the overall feature representation. For the high-level feature $f^{h}$, it can be further represented as:

9 $$f^{h} = [f_{1}^{h} ,f_{2}^{h} ,...,f_{c}^{h} ,]$$

where $f_{i}^{h}$ denotes the feature map of the i-th channel. First, a global average pooling operation is performed on each channel feature $f_{i}^{h}$ to aggregate the information of each feature channel, so as to compress the high-level feature $f_{i}^{h}$ into a C-dimensional feature vector v with the value of its i-th element:

10 $$v_{i} = \frac{1}{W \times H}\sum\limits_{m = 1}^{H} {\sum\limits_{n = 1}^{W} {f_{i}^{h} (m,n)} }$$

where $f_{i}^{h} (m,n)$ denotes the value of position (m, n) on the feature map of the i-th channel. In order to generate the channel attention weights, v is first passed through the fully connected layer and the ReLU activation layer to get $v^{\prime}$, whose dimension becomes C/r in this process, and r is the number of neurons in the fully connected layer. Then the dimension is adjusted to C through the fully connected layer, and finally the attention weights ca are generated for each channel through the sigmoid function. calculated as:

11 $$v^{\prime} = {\text{Re}} LU(fc_{1} v)$$

12 $$ca = sig{\text{mo}} id(fc_{2} v^{\prime})$$

where $fc_{1}$ and $fc_{2}$ denote the corresponding fully connected layers. The final weighted feature output is:

13 $$\tilde{f}^{h} = ca \otimes f^{h}$$

### Classification of emotions

Shallow features usually capture local, detailed, and low-level information, while deep features encapsulate more global, abstract, and high-level semantics. The fusion method helps to improve the accuracy and robustness of the emotion classification system. By combining different feature sets, the system becomes less susceptible to the inherent biases of any single feature type, making it more versatile and reliable across different datasets and scenarios. The ultimate fusion enables the system to identify nuanced emotions that might be overlooked when relying solely on a single feature set.

In order to obtain discriminative visual emotion feature representations, the high and low level features reinforced by the attention mechanism are fused through the feature fusion layer [21], and the input low level features and high level features are firstly subjected to 1X1 convolution operation respectively, to obtain the feature maps $l$ and $h$ with the same size. Then $l$ and $h$ are spliced and fused into discriminative features. Let $f$ denote the discriminative feature obtained after fusion, then $f = l \oplus h$, where $\oplus$ denotes the splicing operation of the feature tensor.

The discriminative feature, obtained after fusing the attentional features, undergoes further processing through a fully-connected layer to generate a one-dimensional semantic vector denoted as d. Subsequently, a softmax classification layer is connected to produce the corresponding probabilities for all sentiment categories, denoted as p. The computational details are outlined as follows:

14 $$p_{i} = \frac{{\exp (d_{i} )}}{{\sum\limits_{i = 1} {\exp (d_{i} )} }},i = 1,...,k$$

where k denotes the emotion category. The cross-entropy loss is used as the loss function for the whole model, defined as follows:

15 $$L = - \sum\limits_{i} {y_{i} \log (p_{i} )}$$

The network can be optimized by minimizing the loss function L through the stochastic gradient descent algorithm. The gradient can be calculated using the following equation:

16 $$\frac{\partial L}{{\partial d_{i} }} = \frac{\partial L}{{\partial p_{i} }}\frac{{\partial p_{i} }}{{\partial d_{i} }} = - \frac{{y_{i} }}{{p_{i} }}(p_{i} (1 - p_{i} )) = p_{i} - y_{i}$$

## Experiments and analysis

In this section, the author conduct a comparative analysis of the proposed DA-MLCNN model and assess its performance on two datasets: Twitter 2017 and Emotion ROI. The Emotion ROI dataset encompasses six emotion categories, namely fear (FEAR), sadness (SADNESS), anger (ANGER), disgust (DISGUST), surprise, and joy. For the experimentation, both datasets are partitioned into 80% for the training set and 20% for the testing set using random division.

### Experimental setup and evaluation indicators

The experimental base network is VGGNet-16, where all convolutional layers employ 3 × 3 convolutional kernels with a step size set to 1, and pooling layers utilize maximum pooling of 2 × 2 with a step size set to 2. The network is pre-trained in ImageNet. The input image is a 224 × 224 RGB color image. To mitigate overfitting, the dataset is augmented by cropping each image sample at 5 positions and randomly flipping it horizontally. The model's batch size for each input is configured to 32.

The optimization of the network is performed using a stochastic gradient descent algorithm, with weight decay set to 0.0005 and a learning rate set to 0.001. To counteract overfitting, the model incorporates the Dropout strategy and the L2 paradigm, with the Dropout value set to 0.5. The experimental development environment consists of Linux-Ubuntu 4.04, Python 2.7, TensorFlow 1.3.0, and PyCharm as the development tool. The training and testing of the model are conducted on a Tesla P100-PCIE GPU workstation.

In this paper, Recall, Precision, F1, and Accuracy are selected as the evaluation metrics for the visual sentiment analysis task to assess the effectiveness of the model, The calculation of each evaluation index is as follows:

16 $$Acc = \frac{TP + TN}{{TP + FP + FN + TN}}$$

17 $$\Pr e = \frac{TP}{{TP + FP}}$$

18 $${\text{Re}} c = \frac{TP}{{TP + FN}}$$

19 $$F1 = \frac{{2*\Pr e*{\text{Re}} c}}{{\Pr e + {\text{Re}} c}}$$

### Performance comparison of visual sentiment analysis with integral and local feature fusion

**Fig. 4:**
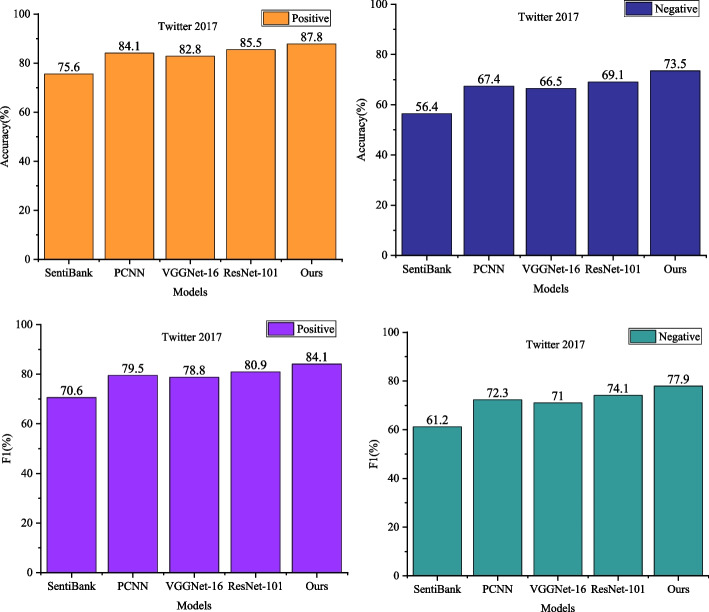
Visual sentiment analysis performance comparison of global and local feature fusion on Twitter 2017 dataset

To assess the visual sentiment analysis performance of the proposed overall and local feature fusion, experiments were conducted with SentiBank [14], PCNN [15], VGGNet-16 [18], and ResNet-101 [22] chosen as comparative models on the Twitter 2017 and Emotion ROI datasets. The Twitter 2017 dataset is a collection of tweets posted on Twitter in the year 2017. This type of dataset has widespread applications in fields such as text analysis, sentiment analysis, topic detection and tracking, and social media monitoring. The Emotion ROI dataset, on the other hand, is a dataset dedicated to image sentiment recognition. It aims to predict or identify the emotions conveyed by images, such as happiness, sadness, anger, and others, through image analysis. Specifically, the results on the Twitter 2017 dataset are presented in Fig. 4.

The proposed method in this paper attains classification accuracies of 87.8% and 73.5% for positive and negative sentiment recognition, marking an average improvement of 7.4% compared to SentiBank, a visual sentiment analysis method based on intermediate semantic representations, and an equivalent average improvement over VGGNet-16, the best-performing deep learning method. Additionally, the proposed method outperforms the ResNet-101 model, the best among deep learning methods, by an average of 4.0%. In terms of the F1 score metric, the proposed method achieves F1 scores of 84.1% and 77.8% for positive and negative sentiment classification, respectively. In contrast, VGGNet-16 achieves F1 scores of 80.9% and 74.1% for positive and negative sentiment classification, highlighting the performance improvement achieved by the proposed method based on VGGNet-16. In comparison to other methods, the visual sentiment analysis with overall and local feature fusion proposed in this paper demonstrates a significant advantage in binary classification on the Twitter 2017 dataset.

**Fig. 5:**
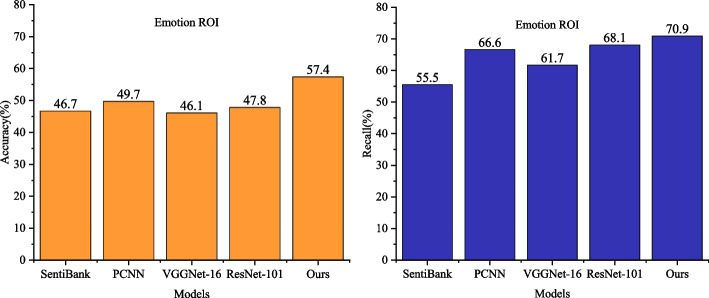
Visual Emotion analysis performance comparison of global and local feature fusion on Emotion ROI dataset

As depicted in Fig. 5, the visual sentiment analysis performance of the proposed method, incorporating the fusion of holistic and local features, is evaluated on the multi-categorization task using the Emotion ROI dataset.

The classification accuracy of the proposed method on the Emotion ROI dataset reaches 57.4%, surpassing that of SentiBank, a visual sentiment analysis method based on intermediate semantic representations. When compared to visual sentiment classification models based on deep learning, specifically VGGNet-16 and ResNet-101, the proposed method exhibits higher accuracy and recall. Against VGGNet-16, the proposed model improves accuracy and recall by 11.3% and 9.2%, respectively. Similarly, when compared to ResNet-101, the proposed model demonstrates improvements of 10.4% in accuracy and 2.8% in recall.

By examining the classification results across various methods on multi-categorization datasets, it is evident that the proposed method is adaptable to the multi-categorization task of visual emotions. The comprehensive performance in both binary and multi-classified emotion image datasets further underscores that the proposed method, leveraging the full utilization of local information, can attain more discriminative visual features, thereby enhancing the overall effectiveness of visual emotion analysis.

### Performance comparison of visual sentiment analysis models under different datasets

To comprehensively validate the effectiveness of the proposed DA-MLCNN model in the realm of sentiment analysis, exhaustive comparative experiments were conducted on the Twitter 2017 dataset. A diverse set of classical methods, including traditional visual sentiment analysis methods, those based on intermediate semantic representations, and deep learning-based methods, were selected for comparison. The specific methods employed for comparison encompass GCH [23], SentiBank [14], VGGNet-16 [18], and COIS [24].

GCH and SentiBank represent traditional visual sentiment analysis methods, relying on manual feature extraction and simple classifiers. In contrast, VGGNet-16 and COIS are deep learning-based sentiment analysis methods capable of automatically learning and extracting complex features from images.

The experimental results showcase that the DA-MLCNN model proposed in this paper achieves a classification accuracy of 79.8% on the Twitter 2017 dataset, outperforming the traditional visual sentiment analysis method, GCH, and the intermediate semantic representation-based method, SentiBank. In comparison to deep learning-based models, DA-MLCNN also exhibits superior performance compared to VGGNet-16 and COIS, with classification accuracies of 75.4% and 78.9%, respectively, on the Twitter 2017 dataset. When compared specifically to the COIS model, DA-MLCNN improves the classification accuracy by 0.93%, a significant enhancement in the field of sentiment analysis.

In summary, the comparative experiments underscore the effectiveness of the DA-MLCNN model in the realm of sentiment analysis. Its classification performance on the Twitter 2017 dataset surpasses all other comparative methods, providing new insights and methodologies for future developments in sentiment analysis.

**Fig. 6:**
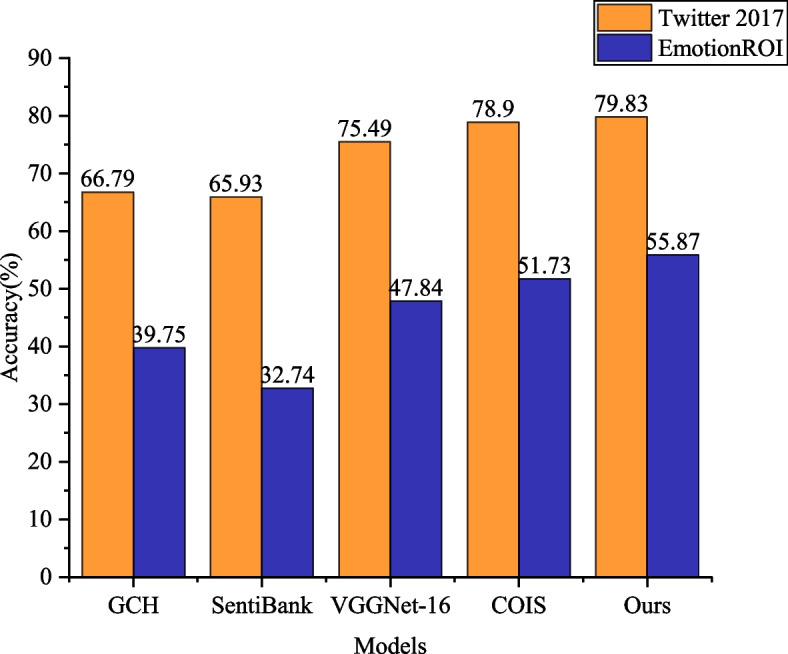
Visual Emotion analysis performance comparison of global and local feature fusion on Emotion ROI dataset

As depicted in Fig. 6, the proposed classification method in this paper demonstrates superior performance on the multi-categorized emotion image dataset Emotion ROI. Achieving a classification accuracy of 55.8%, the method outperforms the VGGNet-16 model by 8.1%. This noteworthy improvement underscores the effectiveness of our model in handling complex emotion recognition tasks.

**Fig. 7:**
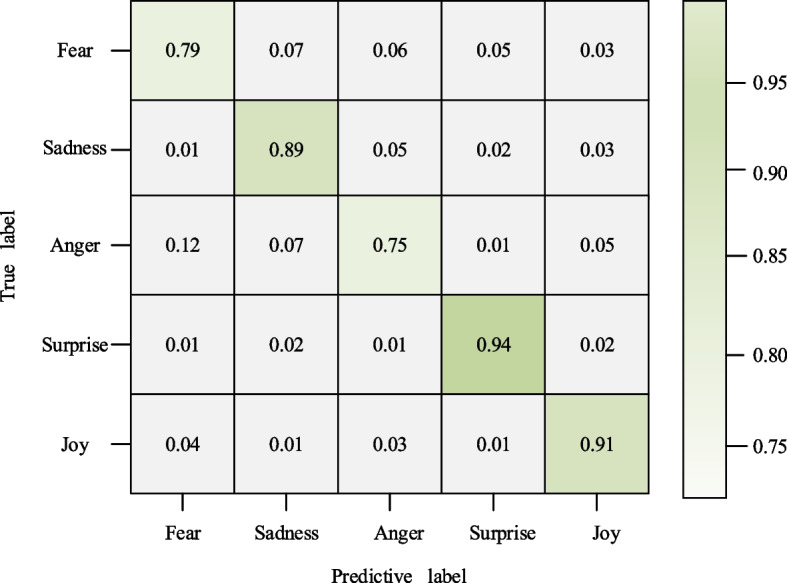
Confusion matrix of different emotion recognition

For a deeper understanding of the model's performance, a detailed analysis is conducted in conjunction with the confusion matrix shown in Fig. 7. The matrix reveals that, particularly for the classification of the three emotion categories of sadness, surprise, and joy, the DA-MLCNN model exhibits remarkably high accuracy rates of 89%, 94%, and 91%, respectively. This implies that our model enables more accurate capturing of users' emotional fluctuations and feedback in visual communication, paving the way for the provision of more personalized services or products.

**Fig. 8:**
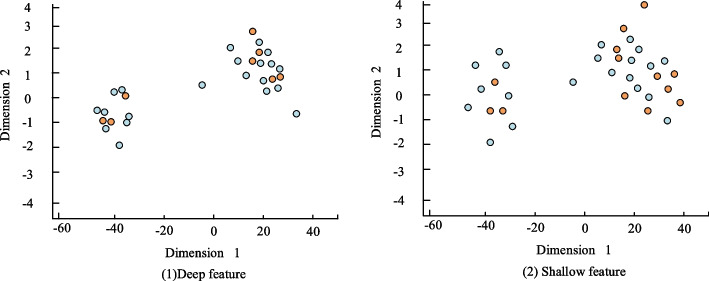
Visual analysis of deep and shallow feature extraction

In the neural network model improved upon VGGNet-16, we are able to capture both shallow and deep features of images through feature extraction at different hierarchical levels, particularly by incorporating multiple convolutional layer branches. Shallow features are extracted from the first or second convolutional blocks of VGGNet-16. As shown in Fig. 8. These features primarily focus on local details of the image, such as low-level information like edges, corners, and textures. Shallow features are highly sensitive to geometric transformations and detail preservation in images. In contrast, deep features are extracted from the fourth and fifth convolutional blocks of VGGNet-16. These features capture more abstract and semantic information of the image, such as object shapes, contours, and categories, which constitute high-level information. Deep features are more sensitive to the global structure and semantic content of the image. By complementing shallow and deep features, we can construct a more robust and accurate visual communication system.

## Discussion

In our experiments, we innovatively integrated multi-layer Convolutional Neural Network (CNN) feature extraction, a dual attention mechanism, and an attention-based feature fusion strategy to propose a cutting-edge visual sentiment analysis method, DA-MLCNN. The core strength of this method lies in its exceptional feature representation capabilities, which deeply mine and fuse both global and local image features, resulting in a richer and more nuanced portrayal of visual content. Specifically, DA-MLCNN employs multi-layer feature fusion techniques to capture diverse information ranging from coarse to fine-grained across different network layers, effectively enhancing the comprehensiveness and depth of feature expression. On this foundation, the introduction of the dual attention mechanism serves as a pivotal enhancement, acting like an intelligent magnifying glass that automatically focuses on key regions and features within images that are closely related to emotional expression, significantly boosting the accuracy and efficiency of sentiment information recognition. This innovative design enables DA-MLCNN to swiftly and accurately pinpoint sentiment cues within vast amounts of data, providing robust technical support for sentiment analysis tasks. Through an adaptive learning and optimization process, DA-MLCNN not only precisely captures subtle emotional nuances but also markedly improves the accuracy and robustness of sentiment classification. Furthermore, we have provided a detailed introduction to the public dataset employed, which serves as the cornerstone for validating the performance of DA-MLCNN. Its richness and diversity ensure the broad applicability and reliability of our experimental results.

The in-depth analysis of visual content by the DA-MLCNN method assists designers in gaining a profound understanding of users' feelings, needs, and behaviors. This understanding aids in better defining design goals and improving the overall user experience. Furthermore, the visual sentiment analysis method based on dual-attention multilayer feature fusion provides designers with more detailed and comprehensive visual information, inspiring innovative ideas. Designers can analyze users' emotions and needs to achieve more personalized designs tailored to different tastes and preferences. The improved performance of DA-MLCNN enhances sentiment analysis accuracy, allowing designers to gain more precise insights into users' emotional needs and preferences. This deep user insight stimulates creative inspiration and innovative design ideas. Accurately analyzing user sentiment enables designers to personalize their designs effectively, strengthening brand loyalty and enhancing the overall user experience.

The performance improvement of the visual sentiment analysis method DA-MLCNN extends its application scope beyond traditional fields like advertising and media. It can now be applied in diverse areas such as movies, games, virtual reality, providing more creative space and opportunities for designers. Accurate sentiment analysis empowers designers to understand users' emotional responses and behavioral patterns, enabling the design of more interactive and engaging products or services. This heightened user interaction enhances user stickiness and loyalty.

## Conclusion

In this paper, the author primarily leverage the important local region information of the image to enhance image representation, harnessing the complementary nature of multilevel features for feature enhancement. The extracted discriminative features are subsequently employed for visual sentiment analysis. A designed convolutional neural network is employed to extract multilevel features from multiple channels of an image. The spatial attention mechanism assigns spatial attention weights to low-level features of multiple channels, while the channel attention mechanism assigns channel attention weights to high-level features of multiple channels, thereby reinforcing feature representations at different levels. To obtain discriminative visual emotion feature representations, the high- and low-level features strengthened by the attention mechanism are fused through the feature fusion layer, forming discriminative features for training emotion classifiers.

The proposed holistic and local feature fusion method achieves notable classification accuracies and F1 values for positive and negative sentiment recognition on the Twitter 2017 dataset. Specifically, it achieves 87.8% and 73.5% classification accuracies, along with 84.1% and 77.8% F1 values for positive and negative sentiment recognition, respectively. Moreover, the final visual sentiment analysis method, DA-MLCNN, demonstrates an accuracy of 89%, 94%, and 91% for categorizing the three categories of sadness, surprise, and joy on the Emotion ROI dataset. The DA-MLCNN method extracts sentiment from visual content, providing designers with emotional feedback about a specific scene or product. This infusion of emotional factors into the design process ensures that designs align more closely with users' psychological feelings and needs, fostering innovation and development in visual communication design.

## Data Availability

No datasets were generated or analysed during the current study.
